# The mediating effect of social functioning on the relationship between social support and fatigue in middle-aged and young recipients with liver transplant in China

**DOI:** 10.3389/fpsyg.2022.895259

**Published:** 2022-08-03

**Authors:** Dan Zhang, Junling Wei, Xiaofei Li

**Affiliations:** ^1^Nursing Department, The First Hospital of China Medical University, Shenyang, China; ^2^Rehabilitation Department, Qingdao Hospital of Traditional Chinese Medicine, Qingdao, China; ^3^Transplantation and Hepatobiliary Department, The First Hospital of China Medical University, Shenyang, China

**Keywords:** social support, social functioning, fatigue, liver transplantation, mediating effect

## Abstract

**Objective:**

The objective of the study was to explore the relationship between social support and fatigue as well as the mediating role of social functioning on that relationship.

**Background:**

Psychosocial factors such as social support and social functioning may influence patients’ fatigue symptoms. There is limited evidence on the relationship between social support, social functioning, and fatigue in liver transplant recipients.

**Methods:**

A total of 210 patients with liver transplants from two tertiary hospitals were enrolled in the current study. Questionnaires used include one for general demographic data, the Perceived Social Support Scale (PSSS), Social Disability Screening Schedule (SDSS), and Fatigue Symptom Inventory (FSI).

**Results:**

A total of 126 (60%) recipients reported fatigue. Gender, residence, BMI, and liver function were the primary factors affecting fatigue. Social support was positively correlated with social functioning and was negatively correlated with fatigue. The effect of social support on fatigue was partially mediated by social functioning (35.74%).

**Conclusion:**

The fatigue of liver transplant recipients should be attended to. The higher the social support, the lower the fatigue of liver transplant recipients. Social support may also reduce fatigue through social functioning. The liver transplant team should help the liver transplant recipient establish a social support system, restore social functioning, and reduce fatigue symptoms.

## Introduction

Liver transplantation (LT) is the most effective method for the treatment of end-stage liver disease. In the past two decades, the number of LT recipients in China has increased, and the survival rate after liver transplantation has improved (Wu, 2018). As a result, concerns for LT recipients have shifted from survival to quality of life. Fatigue is one of the most important indicators of quality of life (van Ginneken et al., 2010).

Fatigue is the most common symptom not only in patients with chronic liver disease (Swain, 2006), but also in LT recipients (Chen et al., 2021). Fatigue often causes great physical and mental distress to LT recipients (Chen et al., 2019). In terms of fatigue time, fatigue symptoms typically do not improve over time; even 15 years after the transplant, 44% of recipients reported severe fatigue (van den Berg-Emons et al., 2006). From the aspect of fatigue type, LT recipients often experience physical fatigue and reduced activity, relatively less mental fatigue, and reduced motivation (Aadahl et al., 2002; van den Berg-Emons et al., 2006). In terms of the impact of fatigue on life, fatigue can lead to poorer health (Kang et al., 2018), increased negative emotional states (Du et al., 2017), a change in normal social functioning (Kang et al., 2018), reduced quality of life (Magalhaes et al., 2017), and may even reduce survival rate (Magalhaes et al., 2017). However, because fatigue is a subjective phenomenon, it can easily be ignored. The conventional wisdom is that you should rest more when you are tired, but this can sometimes backfire because inactivity can lead to muscle atrophy and a loss of heart and lung function, as well as potentially increased psychosocial problems (Timothy and Matthew, 2012), which increases fatigue, creating a cyclical effect. To date, no specific pharmacological strategies have been identified to improve fatigue symptoms (Xu et al., 2017). Among the non-pharmacological interventions, physical activity and psychological therapy remain the primary methods to alleviate fatigue in LT recipients (van den Berg-Emons et al., 2006, 2014; Ergene et al., 2019). Therefore, exploring how to increase physical activity and reduce negative emotions is the key to alleviate fatigue symptoms.

Social functioning is associated with physical activity and psychological symptoms (Chen et al., 2014; Amir et al., 2017). Social functioning is defined as individuals’ interactions with their environment, their ability to fulfill their social roles within their social and community activities, and their relationships with employers, colleagues, friends, partners, and family members (Scoglio et al., 2022). Social dysfunction primarily manifests in a decline of individual interests and social activities, a lack of sense of responsibility and long-term plans, and life skills degradation, among others (Scoglio et al., 2022), which can lead to decreased physical activity and increased negative emotions, such as anxiety and depression (Amir et al., 2017; Zhong, 2020). As far as we know, previous studies have only found that fatigue symptoms affect social functioning in LT recipients (Kang et al., 2018; Walsh et al., 2020). Whether social functioning has an effect on fatigue in the LT population is a question that remains to be elucidated in the literature. According to the theory of unpleasant symptoms (TOUS; Lenz et al., 1997), while symptoms affect bodily functions, they may also be adversely affected by the outcomes of symptoms. Therefore, the current study investigates whether the social functioning of LT recipients influences fatigue. In addition, previous studies have mainly used the SF-36 scale to investigate the social function of LT recipients. Syrjala et al. (2010) indicate that the SF-36 social function scale captures the mental health and perception of interaction components of social health, and not social participation as such. In this study, Social Disability Screening Schedule was used to measure the status of social functioning of LT recipients from the perspective of social participation.

Social support is a multidimensional construction, which refers to the availability of social resources in a specific context (Cipolletta et al., 2019). Different intimate relationships may provide different forms of support, such as psychological and physical support and so on. Studies show that social support is related to social functioning (Tao and Wang, 2010; Qin et al., 2018). Social functioning can be measured by a broad range of structural and functional aspects of social networks. The social network size is an important structural network feature, while receiving social support and the perceived adequacy of support are two important functional aspects of the network (Brenda, 1999; Berkman et al., 2000). A high level of social support may antagonize social dysfunction, which is beneficial to the recovery of social functioning (Tao and Wang, 2010; Qin et al., 2018). Additionally, proper social support can reduce fatigue (Chang et al., 2020; Sorensen et al., 2020; Akbas et al., 2021). Studies have shown that social support is related to anxiety and depression (Fang et al., 2022), anxiety and depression are related to fatigue (Lin, 2016), and anxiety and depression play a mediating role between social support and fatigue (Zhong, 2020). We speculated that social support can reduce psychological fatigue through anxiety and depression, but it is difficult to reduce physical fatigue. Therefore, this study hypothesized that social functioning has a mediating role between social support and fatigue, the following four hypotheses are proposed in the current study: (1) social support will demonstrate a negative effect on fatigue; (2) social support will demonstrate a positive effect on social functioning; (3) social functioning will demonstrate a negative effect on fatigue; and (4) social support will demonstrate a negative and indirect effect on fatigue *via* social functioning.

## Materials and methods

### Design and sampling

The current study was conducted using a cross-sectional design. Between November 2020 and April 2021, LT recipients who fulfilled the inclusion criteria of the study at the First Affiliated Hospital of China Medical University and the Tianjin First Central Hospital were recruited as study subjects. The inclusion criteria included: (1) having received a primary LT; (2) recipients aged between 18 and 65 years; (3) normal comprehension; and (4) 6 months or more post-LT. The exclusion criteria included any severe psychiatric or cognitive diseases.

### Questionnaires

#### Demographic and clinical characteristics

Demographic information included gender, age, residence, marital status, body mass index (BMI), *per capita* monthly income (CNY), economic burden, and employment status. Clinical characteristics included the etiology of liver disease and liver function.

#### Measurement of social support

The Perceived Social Support Scale (PSSS) was designed by Zimet et al. (1990) in 1988 to measure the perceived individual social support. It includes three dimensions, namely family support, friend support, and other support. Each of the 12 items is scored from 1 (totally disagree) to 7 (totally agree). The total score ranges from 12 to 84, and the score is related to the level of social support. The scale has been demonstrated to have good validity and reliability in Chinese groups (Lin, 2016). The Cronbach’s alpha is 0.934 in the current study.

#### Measurement of fatigue

The Fatigue Symptom Inventory (FSI) was developed by Hann et al. (1998) in 1998 to measure patients’ fatigue during the past week. Yang et al. (2007) sinicized the scale for use in a Chinese population. The FSI consists of 13 items that assess fatigue intensity (4 items), fatigue duration (2 items), and the degree to which fatigue affects life (7 items). Fatigue intensity refers to the highest, lowest, and average levels of fatigue over the past week as well as the current level of fatigue (0 = no fatigue at all, 10 = extreme fatigue). A composite fatigue intensity score is obtained by averaging the intensity items. Frequency was assessed for the number of days in the week prior to the assessment (0–7 days) and the amount of time each day fatigue was present (0 = none of the day, 10 = all day). Perceived interference was assessed to ascertain the extent to which fatigue in the previous week interfered with general activity levels, the ability to bathe and dress, work activities, the ability to concentrate, relationships with others, enjoyment of life, and mood (0 = no interference, 10 = extreme interference). The score of this dimension is calculated by taking the sum of the average scores of the seven items. Adding the scores of these three dimensions provides a total fatigue score. A higher score suggests higher levels of fatigue. A score of three or greater has been established as the cutoff for identifying clinically significant fatigue. The FSI has good reliability and validity in Chinese groups (Lin, 2016) and the Cronbach’s α alpha is 0.966 in the current study.

#### Measurement of social functioning

The Social Disability Screening Schedule (SDSS) was developed by the World Health Organization (WHO) to assess patients’ social functioning (Ma, 2002). There are 10 items in total, including occupation and work, marital function, parental function, social withdrawal, social activities outside of the family, activities within the family, family function, self-care in daily life, responsibility and planning, and interest and attention in the environment. Each item is divided into three grades; 0 = normal, 1 = partial social dysfunction, and 2 = serious social dysfunction. The total score is the sum of the score of 10 items. A total score ≥2 was considered to have social dysfunction. The scale has good reliability and validity in Chinese patients (Qin et al., 2018) and, in the current study, Cronbach’s alpha is 0.786.

#### Data collection procedure

Data were gathered using face-to-face interviews. Informed consent was obtained prior to the distribution of the questionnaire. To ensure the authenticity of the study, the purpose, significance, and confidentiality of the study were explained to the participants. A total of 210 valid questionnaires were collected, with a validity rate of 96.3%. This study was conducted according to the guidelines provided in the Declaration of Helsinki, and all of the procedures were approved by the ethics committee of the research center.

#### Statistical analysis

Statistical analyses were conducted using SPSS version 25.0. All tests were two-sided and *p* < 0.05 was set as statistically significant. The distribution of fatigue among demographic and clinical characteristics was analyzed with non-parametric tests. According to the univariate analysis (*p* < 0.10) and clinical relevance (Akbulut et al., 2020), seven variables were selected as control variables in the multivariate hierarchical model. Correlations betwen social support, social functioning, and fatigue were calculated using Spearman correlations. A hierarchical multiple regression analysis was used to test social functioning as a potential mediating player in the relationship between social support and fatigue. First, control variables were input (Block 1). Second, social support was added (Block 2). Finally, social functioning was added as a mediator (Block 3). To further assess the mediating role of social functioning, 5,000 bootstrap samples were used *via* the PROCESS macro. If the BCa 95% confidence interval (CI) does not contain 0, there is determined to be a significant mediating effect.

## Results

### Participant characteristics

Among the 210 LT recipients, the mean age was 49.56 ± 8.24 (20–65 years). Table 1 shows the demographic and clinical characteristics of the sample population as well as the results of the univariate model. Gender, residence, BMI, economic burden, liver function, and etiology of liver disease were all related to fatigue (*p* < 0.10) and were therefore entered as control variables into the hierarchical multiple linear regression model.

**Table 1:** Demographic and clinical characteristics by fatigue and results of the univariate.

Variables	Fatigue		Z	*P*
	*N*(%)	Median (IQR)		
Gender			−1.25	0.210
Female	36(17.14)	11.95(11.96)		
Male	174(82.86)	11.09(12.30)		
Age			−0.46	0.644
<40	28(13.3)	11.89(14.27)		
40–65	182(86.7)	11.20(11.57)		
Residence			−3.08	0.002
Rural	34(16.19)	19.34(15.15)		
Urban	176(83.81)	11.00(10.38)		
Marital status			−1.61	0.108
Single/Divorced/Widowed	14(6.67)	16.07(15.57)		
Married	196(93.33)	11.13(11.77)		
BMI			−2.03	0.043
Abnormal	91(43.33)	12.57(14.50)		
Normal	119(56.67)	10.96(11.11)		
Economic burden			7.13	0.068
No	24(11.43)	6.89(10.34)		
Light	56(26.67)	9.71(9.71)		
Medium	72(34.29)	13.09(11.52)		
Heavy	58(27.62)	12.68(14.49)		
Etiology of liver disease			8.31	0.040
Viral cirrhosis	72(34.29)	8.95(11.65)		
Alcoholic cirrhosis	19(9.05)	13.18(15.32)		
Hepatocarcinoma	69(32.86)	13.00(10.63)		
Other disease	50(23.81)	10.20(11.73)		
Liver function			−2.56	0.011
Abnormal	26(12.38)	11.46(11.42)		
Normal	184(87.62)	10.63(11.96)		
Complication			−1.04	0.299
Yes	135(64.29)	11.89(12.36)		
No	75(35.71)	11.14(12.36)		
*Per capita* monthly income (CNY)			0.14	0.934
<=2,000	32(15.24)	12.50(14.95)		
2,000–4,000	72(34.29)	11.68(10.04)		
> = 4,000	106(50.48)	10.95(12.65)		

IQR (Q3-Q1): Inter-quartile range.

### Correlations between social support, social functioning, and fatigue

Table 2 shows that social support and social functioning were both correlated with fatigue. Social support was negatively correlated with fatigue (*r* = −0.35, *p* < 0.01) and social dysfunction (*r* = −0.34, *p* < 0.01). Furthermore, social dysfunction was positively associated with fatigue (*r* = 0.29, *p* < 0.01).

**Table 2:** Means, SDs, and correlations of all variables.

Variables	Mean	SD	Social support	Fatigue
Social support	67.18	12.73	1	
Fatigue	11.72	7.62	−0.35^**^	1
Social functioning	2.21	2.55	−0.34^**^	0.29^**^

SD, standard deviation.

** *p* < 0.01 (two-tailed).

### The results of the hierarchical multiple regression

As shown in Table 3, social support explained 9% and social functioning explained 5% of the variance in fatigue. Social support was negatively correlated with fatigue (*β* = −0.14, *p* < 0.01) and social dysfunction was positively correlated with fatigue (*β* = 0.72, *p* < 0.01). When social dysfunction was added in step 3, the absolute value of the regression coefficient of social support on fatigue was decreased from −0.18 to −0.14, suggesting that social functioning may act as a mediator between social support and fatigue in LT recipients.

**Table 3:** Hierarchical multiple linear regression analysis results.

Variables	Fatigue
	Step 1 (*β*)	Step 2 (*β*)	Step 3 (*β*)
Block 1			
Gender	−2.40	−2.14	−2.60^*^
Residence	−4.09^**^	−4.26^**^	−3.23^**^
BMI	−2.42^*^	−2.01^*^	−2.01^*^
Economic burden	0.91	0.30	0.24
Liver function	−4.84^**^	−4.04^**^	−3.31^*^
Etiology of liver disease	0.15	0.14	0.24
Block 2			
Social support		−0.18^**^	−0.14^**^
Block 3			
Social functioning			0.72^**^
R2	0.15	0.24	0.28
△R2	0.15	0.09	0.05

* *p* < 0.05.

** *p* < 0.01(two-tailed).

### The results of the path analysis

**Figure 1:**
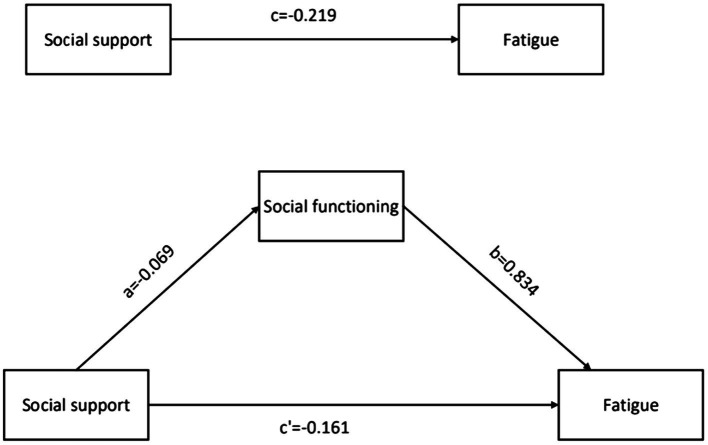
The hypothesised mediation model relating the effect of social support on fatigue through social functioning. Indirect effect of social support on fatigue through social functioning = −0.057 (*p* < 0.01, 95% confidence interval, −0.105 to −0.022).

Path coefficients are shown in Figure 1. First, the total effect of social support on fatigue (path c) was −0.218 (*p* < 0.01). The coefficients of path a were − 0.069 (*p* < 0.01), path b was 0.834 (*p* < 0.01), and path c’ was −0.161 (*p* < 0.01), which suggests that social support, social functioning, and fatigue are independently related. Furthermore, the indirect effect of social support on fatigue through social dysfunction was −0.057 (*p* < 0.01, 95% CI: −0.105, −0.022), which suggests that the relationship of social support on fatigue (a × b) was partly mediated by social functioning. Finally, to understand the effect size of the mediating pathway, we used the formula (a × b)/c to calculate the proportion of the indirect effect of social functioning accounting for the total effect of social support on fatigue, with the resulting mediating effect of 35.74%.

### Intensity, duration, and interference of fatigue

The fatigue status is shown in Table 4. Mean scores of fatigue intensity items were 4.84 ± 2.65 (most fatigue), 2.86 ± 2.55 (least fatigue), and 3.62 ± 2.15 (average fatigue) in the week prior to the assessment and 2.96 ± 2.29 at the point of assessment. The number of days fatigued in the week prior to the assessment was 2.24 ± 1.87 and the average amount of time fatigued each day was 3.04 ± 2.36. The mean score of fatigue interference was 2.87 ± 2.08. The fatigue interference scores from high to low included general activity level, enjoyment of life, work activity, mood, ability to concentrate, relationships with others, and the ability to bathe and dress.

**Table 4:** Liver transplant recipients’ scores on the FSI.

	Min	Max	Mean	SD
Intensity ratings (subscale score)			3.57	2.03
Most fatigue	0	10	4.84	2.65
Least fatigue	0	10	2.86	2.55
Average fatigue	0	9	3.62	2.15
Fatigue at the point of assessment	0	10	2.96	2.29
Duration ratings (subscale score)			5.28	4.07
Number of days fatigued	0	9	3.18	2.24
Amount of time fatigued	0	10	2.40	2.18
Interference scale (subscale score)			2.87	2.08
General activity level	0	10	2.93	2.28
Ability to bathe and dress	0	9	2.89	2.18
Work activity	0	9	2.80	2.20
Ability to concentrate	0	9	2.99	2.37
Relations with others	0	9	2.89	2.36
Enjoyment of life	0	7	2.24	1.87
Mood	0	10	3.04	2.36

FSI, Fatigue Symptom Inventory; SD, standard deviation.

## Discussion

The current study is the first to explore the relationship between social support, social functioning, and fatigue in middle-aged and young recipients with LT and confirms that social functioning plays a partial mediating role in the relationship between social support and fatigue. A total of 126 (60.0%) of the 210 LT recipients reported fatigue (average fatigue intensity score ≥3). This result is consistent with previous studies (66–76%; van den Berg-Emons et al., 2006; James et al., 2010). Also consistent with previous studies, fatigue interfered most with general activity levels and least with the ability to bathe and dress (James et al., 2010; Lin et al., 2017). These demonstrate that fatigue caused problems in most of the recipients and should be paid attention to.

The final model demonstrated that, consistent with previous research (Karadag et al., 2013; Lin, 2016; Sorensen et al., 2020; Akbas et al., 2021), social support was negatively correlated with fatigue; that is, the higher the level of social support, the lower the fatigue. Karadag et al. (2013) showed that among hemodialysis patients, patients with higher social support had lower fatigue. Akbas et al. (2021) suggested that social support for breast cancer patients is important for both physical and mental fatigue. On the one hand, good social support helps patients establish positive self-management and compliance behaviors (Zhang et al., 2022a), promotes active cooperation with treatment, and reduces the occurrence of fatigue symptoms. On the other hand, more social support means more available coping resources such as financial support and emotional support, which can enhance patients’ self-confidence and self-efficacy (Aune et al., 2021; Banik et al., 2021), thereby aiding patients in actively dealing with the symptoms of the disease and proactively seeking and obtaining ways to alleviate fatigue symptoms. In addition, good social support has been shown to improve sleep quality in LT recipients (Xu et al., 2021; Zhang et al., 2022b), thereby reducing symptoms of fatigue (Lin et al., 2017).

Our study is the first to include and reveal that good social functioning can alleviate fatigue symptoms. There is a dearth of studies on the relationship between them. Syrjala et al. (2010) showed that fatigue was a significant predictor of social functioning, but they also stressed that the cross-sectional design could only show an association between the two, not that fatigue caused social functioning. Our study made up for the lack of cross-sectional studies and enriched the research on the correlation between social functioning and fatigue. The current existing studies have shown physical activity and psychological wellness can reduce fatigue symptoms (Chen et al., 2014; van den Berg-Emons et al., 2014; Amir et al., 2017; Ergene et al., 2019; Yu et al., 2020). Therefore, we deem that the reason why good social functioning can reduce fatigue may be through increasing physical activity and alleviating psychological problems.

More importantly, social support not only has a direct effect on fatigue, but it also has an indirect effect through social functioning. The higher the social support, the better the social functioning (Tao and Wang, 2010; Qin et al., 2018; Willard et al., 2020). Husson et al. (2017) suggested that strengthening social support may help young cancer survivors better reintegrate into society. Victoria demonstrated that good social functioning is related to a high level of social support in adolescent and young adult survivors of childhood cancer (Willard et al., 2020). A possible reason for this is that higher levels of social support may improve social functioning by promoting social contact and emotional communication, thereby reducing fatigue. These are potential explanations of the association between social support, social functioning, and fatigue. Therefore, this study provides a new direction for the intervention of fatigue in middle-aged and young recipients with LT by increasing social support and improving social functioning.

## Conclusion

In conclusion, the fatigue experienced by LT recipients should be given attention. The current study shows that gender, residence, BMI, and liver function are the primary factors affecting fatigue. Additionally, as social support scores rise, fatigue scores in LT recipients tend to drop. Social support can also reduce fatigue through social functioning. This finding has important implications for both the management as well as the prevention of fatigue symptoms in LT recipients. The management of fatigue should not be limited to pathophysiological considerations, but should also focus on psychosocial needs. The LT team, the recipient’s family, and the community should all help the LT recipient establish a social support system to encourage their reintegration into society, for example, through active employment and participation in social activities, to restore social functioning, and thereby reduce fatigue symptoms.

## Limitations

The current study has some limitations. First, the variables in this study explained only 28% of the total variance of fatigue symptoms, suggesting that the following studies could investigate fatigue in LT recipients from other potential variables and intervene from a psychosocial perspective. Second, although this study is a multi-center study, the sample size is limited, so we need to confirm and generalize the current research results by more representative samples. Third, this is a cross-sectional study, further longitudinal studies are needed to determine causal the relationship between the study variables.

## Data availability statement

The raw data supporting the conclusions of this article will be made available by the authors, without undue reservation.

## Author contributions

XL and DZ designed the study. DZ collected the data. DZ and JW analyzed the data and drafted the manuscript. XL revised the manuscript. All authors read and approved the final manuscript.

## Conflict of interest

The authors declare that the research was conducted in the absence of any commercial or financial relationships that could be construed as a potential conflict of interest.

## Publisher’s note

All claims expressed in this article are solely those of the authors and do not necessarily represent those of their affiliated organizations, or those of the publisher, the editors and the reviewers. Any product that may be evaluated in this article, or claim that may be made by its manufacturer, is not guaranteed or endorsed by the publisher.
